# Soluble Human Epidermal Growth Factor Receptor 2 (sHER2) as a Potential Risk Assessment, Screening, and Diagnostic Biomarker of Lung Adenocarcinoma

**DOI:** 10.3390/diagnostics3010013

**Published:** 2013-01-14

**Authors:** Abby L. Cosentino-Boehm, Jacqueline M. Lafky, Tammy M. Greenwood, Kimberly D. Kimbler, Marites C. Buenafe, Yuxia Wang, Adam J. Branscum, Ping Yang, Nita J. Maihle, Andre T. Baron

**Affiliations:** 1 Department of Preventive Medicine, Northwestern University Biomedical Informatics Center, NUCATS 750 N. Lake Shore Dr., 11th Floor, Chicago, IL 60611, USA; E-Mail: a-cosentino-boehm@northwestern.edu; 2 Mayo Clinic Cancer Center, Mayo Clinic, 200 First Street S.W., Rochester, MN 55905, USA; E-Mails: lafky.jacqueline@mayo.edu (J.M.L.); greenwood.tammy@mayo.edu (T.M.G.); 3 Department of Obstetrics and Gynecology, Division of Gynecologic Oncology, University of Kentucky, Lucille P. Markey Cancer Center, Lexington, KY 40536, USA; E-Mail: kdkimb2@email.uky.edu; 4 Department of Family Medicine, University of Kentucky, College of Medicine, 800 Rose Street, Lexington, KY 40536 ,USA; E-Mail: marites.buenafe@eku.edu; 5 University of Massachusetts Medical School, 55 Lake Avenue North, Worcester, MA 01655, USA; E-Mail: wyerliang@yahoo.com; 6 School of Biological and Population Health Sciences, Oregon State University, Corvallis, OR 97331, USA; E-Mail: adam.branscum@oregonstate.edu; 7 Department of Health Sciences Research, Mayo Clinic, 200 First Street S.W., Rochester, MN 55905, USA; E-Mail: yang.ping@mayo.edu; 8 Department of Obstetrics, Gynecology and Reproductive Sciences, Yale University School of Medicine, P.O. Box 2068063, New Haven, CT 06520 ,USA; E-Mail: nita.maihle@yale.edu; 9 Department of Epidemiology, University of Kentucky, College of Public Health, 111 Washington Avenue, Lexington, KY 40536, USA

**Keywords:** soluble human epidermal growth factor receptor 2 (sHER2), non-small cell lung cancer, adenocarcinoma, squamous cell carcinoma (SCC), risk assessment, screening, early detection, diagnosis

## Abstract

Lung cancer is the leading cause of cancer-related death in the United States. Here, we evaluated the potential clinical utility of soluble human epidermal growth factor receptor 2 (sHER2) for the risk assessment, screening, and diagnosis of non-small cell lung cancer (NSCLC) using an unmatched case-control study design. Serum sHER2 concentrations were measured by immunoassay in 244 primary NSCLC cases and 218 healthy controls. Wilcoxon rank-sum tests, logistic regression models, and receiver operating characteristic plots were used to assess whether sHER2 is associated with lung cancer. Median serum sHER2 concentrations are higher in patients with adenocarcinoma than squamous cell carcinoma regardless of gender, and sHER2 is a weak, independent biomarker of adenocarcinoma, but not of squamous cell carcinoma, adjusted for age and gender. The age-adjusted relative risk (odds) of adenocarcinoma is 3.95 (95% CI: 1.22, 12.81) and 7.93 (95% CI: 2.26, 27.82) greater for women and men with high sHER2 concentrations (≥6.60 ng/mL) *vs.* low sHER2 concentrations (≤1.85 ng/mL), respectively. When adjusted for each other, sHER2, age, and gender discern healthy controls from patients with primary adenocarcinomas of the lung with 85.9% accuracy. We conclude that even though serum sHER2 is not a strong, stand-alone discriminatory biomarker of adenocarcinoma, sHER2 may be a useful, independent covariate in multivariate risk assessment, screening, and diagnostic models of lung cancer.

## 1. Introduction

Lung cancer is the leading cause of cancer-specific mortality for men and women in the U.S. [1,2,3]. The National Cancer Institute’s (NCI) Surveillance Epidemiology and End Results program estimated that 226,160 new lung cancer cases were diagnosed in the U.S. in 2012, and that 160,340 individuals died of this disease. Of all lung cancers, 99% consist of either small cell carcinomas (13.9%) or non-small cell carcinomas (85.1%). Histological subtypes of non-small cell lung cancer (NSCLC) include adenocarcinomas (37.5%), squamous and transitional cell carcinomas (19.8%), large cell carcinomas (3.3%), and other tumor subtypes (24.5%). Patients diagnosed with localized, early stage lung cancer have a 5-year survival rate of 49.5% and may be cured by surgical resection [4,5]. However, 77% of lung cancer patients have advanced stage disease at diagnosis, and their 5-year survival rate is only 20.6% for disease that has spread to the regional lymph nodes or 2.8% for disease that has metastasized to distant anatomical sites. Because patients who are diagnosed with early stage lung cancer have a significantly better prognosis, screening (*i.e*., early detection) represents a practical public health approach for decreasing lung cancer mortality.

Throughout the 1960s and 1970s, chest x-ray (CXR) alone or in conjunction with sputum cytology were evaluated to detect early stage lung cancer [4,5]. To assess the feasibility, validity, and efficacy of CXR for lung cancer screening, the NCI sponsored the Prostate, Lung, Colorectal, and Ovarian (PLCO) randomized screening trial, which was designed with a “no-screening” study arm and 89% power to detect a 10% reduction in lung cancer mortality [6,7]. While awaiting PLCO trial results [8,9], contemporary researchers have focused on developing low dose computed tomography and biomarkers as potential lung cancer screening modalities [10,11,12,13,14,15,16].

The human epidermal growth factor receptor 2 (*HER2/ERBB2/neu*) proto-oncogene encodes a cell surface receptor tyrosine kinase (RTK) that functions to regulate cell proliferation and survival [17]. *HER2* amplification and protein overexpression have been implicated in the etiology and pathogenesis of several human malignancies including lung cancer [18]. HER2 is overexpressed in 10–20% of NSCLC cases and in perhaps as many as 30% of lung adenocarcinomas [19,20,21,22,23], where overexpression is associated with adverse tumor characteristics and poor patient prognosis [24,25]. In addition to full-length HER2, cells synthesize “soluble” HER2 (sHER2) isoforms [26,27,28,29,30]. These sHER2 isoforms are produced either by alternate mRNA splicing or by proteolytic cleavage, and, are either secreted or proteolytically shed from the plasma membrane into extracellular body fluids. Alternate splicing results in mRNA transcripts that encode 68-kDa [30] and 100-kDa [28] sHER2 isoforms; whereas, proteolytic cleavage results in 105-kDa [26] and 110-kDa [27] shed isoforms of sHER2 that encompass only extracellular subdomains of this RTK. While it is widely assumed that the 105-kDa sHER2 isoform represents the major constituent of human blood, a careful biochemical characterization of serum sHER2 isoforms has not yet been performed. Nonetheless, multiple assays to quantify sHER2 have been developed and used to assess the potential clinical utility of serum sHER2 in cancer patients across disease sites [31,32]. In particular, serum sHER2 has been examined in preliminary studies of lung cancer, but has not yet been rigorously validated as a potential risk assessment, screening, and/or diagnostic biomarker. Initial reports suggest that serum sHER2 is elevated in 5–64% of lung cancer cases [23,32,33,34,35,36] and may be elevated many months prior to clinical diagnosis [33]. 

In this study, we evaluated 244 primary NSCLC cases and 218 healthy controls using an unmatched, retrospective, case-control study design to determine whether age and/or gender are confounders or effect modifiers of the relationship between sHER2 and NSCLC. We observed that sHER2 concentrations are associated with age among healthy men, and differ between healthy men and women. Although sHER2 concentrations do not differ between healthy controls and patients with NSCLC overall, they are slightly higher in patients with adenocarcinoma regardless of gender, but not squamous cell carcinoma. Logistic regression models further demonstrate that sHER2 is a weak, independent classifier of adenocarcinoma but not of squamous cell carcinoma, and when adjusted for age and gender, the risk of adenocarcinoma increases with higher sHER2 concentrations. Moreover, when mutually adjusted for each other, sHER2, age, and gender distinguish healthy controls from patients with adenocarcinoma with 85.9% accuracy. These data suggest that albeit serum sHER2 is not a strong, stand-alone discriminatory classifier of adenocarcinoma, its independence of age and gender may confer some limited utility to sHER2 as a covariate in multivariate models for the risk assessment, screening, and/or diagnosis of lung adenocarcinoma.

## 2. Materials and Methods

### 2.1. Serum Samples

Serum samples were collected at the Mayo Clinic, Olmsted County, Rochester, MN, and stored at −80 °C, as described in detail previously, from 218 healthy controls between 1981 to 1984, and 244 primary NSCLC cases between 1997 to 2002 through a “Normal Values Study” [37] and “Comprehensive Lung Cancer Resource” [38], respectively; both protocols were approved by the Mayo Clinic Institutional Review Board. Written informed consent was obtained from each participant and all biospecimens were redacted of the patient’s identity. Each control was annotated with age and, if female, menopausal status at venipuncture; and each lung cancer case was annotated with age, tumor histological subtype, and, if female, menopausal status at diagnosis. Information concerning disease stage and tumor grade were unavailable. The cases included 79 patients with squamous cell carcinoma (SCC) and 165 with adenocarcinoma.

### 2.2. sHER2 ELISA

Serum sHER2 concentrations were measured using the ^sp185^HER-2 ELISA (Bender MedSystems Diagnostics GmbH, Vienna, Austria) according to the manufacturer’s instructions. The manufacturer reports an analytical detection limit of 0.06 ng/mL sHER2 (two standard deviations above the mean absorbance observed with sample buffer), mean intra-assay coefficient of variation of 1.9%, mean inter-assay coefficient of variation of 5.8%, and mean spike recovery of 89% with serum for this ELISA. In addition, the manufacturer reports no significant loss of serum sHER2 immunoreactivity after five freeze-thaw cycles at −20 °C or following a 24 h storage period at −20 °C, 2–8 °C, 24 °C, or 37 °C, indicating that sHER2 is not an inherently unstable serum protein. 

All serum samples in the present study were initially quantified in quadruplicate at a 1:20 dilution. Serum samples yielding absorbance values outside the linear range of the assay’s standard curve, below the biological detection limit (four standard deviations above the mean absorbance observed with sample buffer), or with a coefficient of variation ≥20% were re-assayed at either a 1:10, 1:40, 1:80, or 1:120 dilution to obtain accurate estimates of serum sHER2 concentrations. We report a mean inter-assay analytical detection limit of 0.02 ng/mL, mean inter-assay biological detection limit of 0.05 ng/mL, mean intra-assay coefficient of variation of 8.27% and 8.45% with normal serum sample controls estimated to contain 3.2 and 7.7 ng/ml sHER2, respectively, and mean inter-assay coefficient of variation of 4.11% and 2.26% with low and high quality control standards supplied by the manufacturer estimated to contain 20.9 and 146.6 ng/ml sHER2, respectively.

### 2.3. Statistical Analysis

Statistical analyses were performed using “R” (R Foundation for Statistical Computing, Vienna, Austria. http://www.R-project.org) and SAS version 9.2 (SAS Institute, Cary, NC, USA). Descriptive statistics were calculated, and the nonparametric Wilcoxon rank-sum test was used to determine whether sHER2 concentrations differ significantly between healthy controls and patients with primary NSCLC, before and after stratification by gender, tumor histology, and menopausal status. Nonparametric local weighted regression and Spearman’s rank-order correlation coefficients (ρ) were calculated to determine whether associations exist between sHER2 concentrations and age for healthy controls or patients with NSCLC by gender. Univariate and multivariate logistic regression analyses were performed to assess whether log-transformed sHER2 concentrations, age, and gender are associated with lung cancer. Logistic regression models were compared for their ability to discriminate NSCLC from control patients and adenocarcinoma from SCC tumors using the area under the curve (AUC) of receiver operating characteristic (ROC) plots, and to assess the age- and gender-adjusted effect of sHER2 on cancer risk. Statistical adjustment for multiple comparisons was performed with the step-up procedure developed by Benjamini and Hockberg (1995) to control the false discovery rate (FDR) [39,40]. FDR p-values <0.05 differ significantly between diagnostic groups; however, because this is an exploratory hypothesis generating study of a small sample size, after stratification by gender and tumor histological subtype, FDR p-values between 0.10 and 0.05 were considered to be of borderline significance.

## 3. Results

### 3.1. sHER2 Concentrations Are Associated with Age in Healthy Men

**Figure 1:**
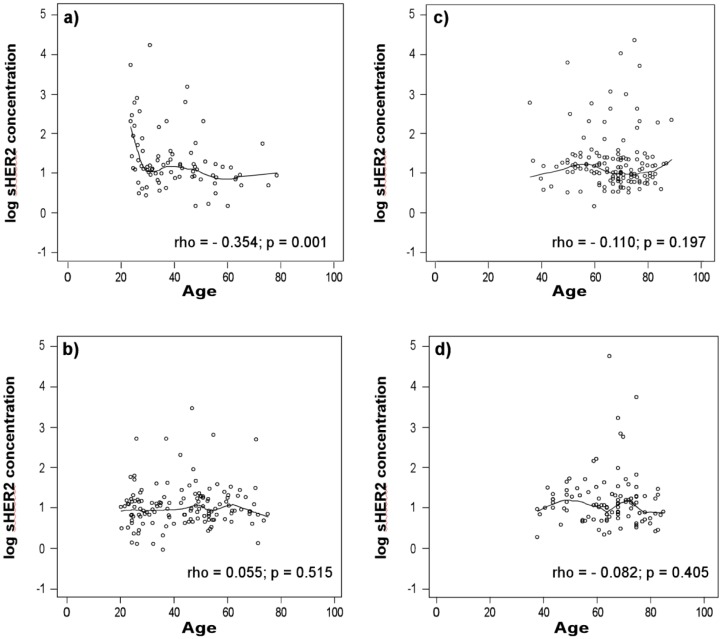
Log soluble human epidermal growth factor receptor 2 (sHER2) concentrations *vs.* age. Log-transformed serum sHER2 concentrations are plotted against age (years) with nonparametric regression curves for healthy men (**a**), men with non-small cell lung cancer (**b**), healthy women (**c**), and women with non-small cell lung cancer (**d**). Spearman correlations (rho) and p-values are given for each comparison.

This unmatched, retrospective, case-control study consists of 218 healthy controls (81 men, 137 women) and 244 patients with primary NSCLC (139 men, 105 women). The healthy men and women in this study are younger (median age: 47 and 38 years, respectively) than the men and women with NSCLC (median age: 68 and 64 years, respectively, p < 0.0001; FDR p < 0.0005, Table 1). Since the cases and controls are unbalanced with regard to both gender and age, these parameters represent potential confounders or effect modifiers of the association between serum sHER2 concentrations and a classification of NSCLC. Therefore, we assessed whether sHER2 is associated with age among healthy participants or patients with NSCLC in this study population by gender (Figure 1). These analyses show that sHER2 concentrations are not associated with age among healthy women (Figure 1(B); ρ = 0.055; p = 0.515; FDR p = 0.649), women with NSCLC (Figure 1(D); ρ = −0.082; p = 0.405; FDR p = 0.537), or men with NSCLC (Figure 1(C); ρ = −0.110; p = 0.197; FDR p = 0.298). In contrast, sHER2 concentrations decrease with age in healthy men (Figure 1(A); ρ = −0.354; p = 0.001; FDR p = 0.004). Furthermore, sHER2 concentrations do not differ between healthy premenopausal *vs.* postmenopausal women (p = 0.731; FDR p = 0.824), or between healthy premenopausal (p = 0.966; FDR p = 0.966) and postmenopausal (p = 0.102; FDR p = 0.169) women *vs.* women with NSCLC of the same menopausal status (Table 1). Stratification of NSCLC cases by histological subtype further shows that sHER2 concentrations are not associated with age among all patients with SCC (ρ = −0.045; p = 0.692; FDR p = 0.815) or adenocarcinoma (ρ = −0.063; p = 0.421; FDR p = 0.544), men with SCC (ρ = −0.095; p = 0.530; FDR p = 0.653) or adenocarcinoma (ρ = −0.113; p = 0.281; FDR p = 0.414), and women with SCC (ρ = −0.008; p = 0.963; FDR p = 0.966) or adenocarcinoma (ρ = −0.042; p = 0.726; FDR p = 0.824).

**Table 1:** Comparison of age and serum concentrations of soluble human epidermal growth factor receptor 2 (sHER2) in healthy controls *vs.* lung cancer cases. Age and serum sHER2 concentrations are compared between healthy men and women (*i.e*., controls) *vs.* patients with lung cancer by gender, and by menopausal status among healthy women.

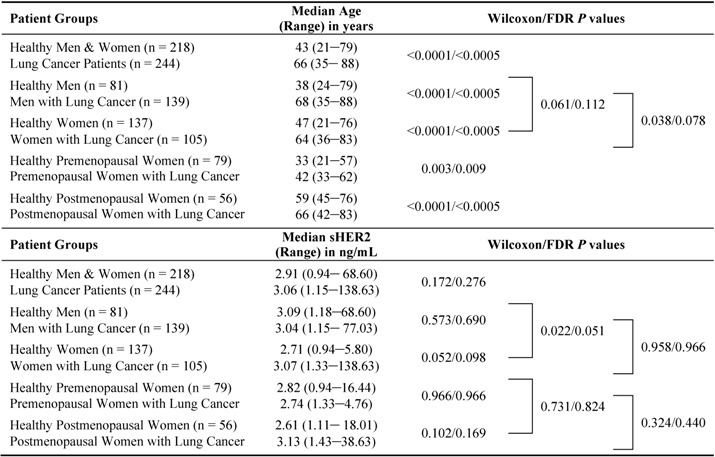

### 3.2. sHER2 Concentrations are Higher in Patients with Adenocarcinoma

**Figure 2:**
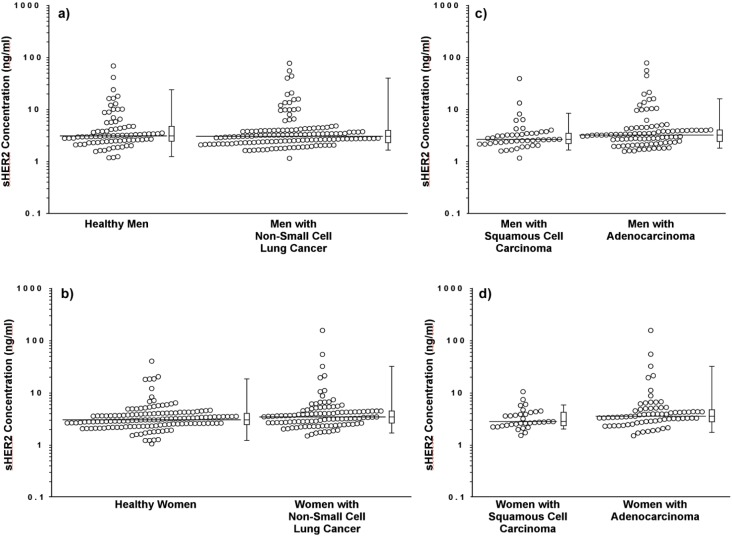
Scattergrams of sHER2 concentrations in healthy controls and patients with lung cancer. Serum sHER2 concentrations are compared between healthy men *vs.* men with non-small cell lung cancer (**a**), healthy women *vs.* women with non-small cell lung cancer (**b**), men with squamous cell carcinoma (SCC) *vs.* adenocarcinoma (**c**), and women with SCC *vs.* adenocarcinoma (**d**). Each data point represents the median sHER2 concentration for one serum sample assayed in quadruplicate. The horizontal lines indicate the median serum sHER2 concentration for each group of participants. Horizontal lines in the box plot represent the first, second (median), and third quartiles; whiskers extend from the box to a distance of 1.5 interquartile ranges.

Although sHER2 concentrations do not differ between healthy controls *vs.* patients with NCSLC (Table 1; p = 0.172; FDR p = 0.276), men *vs.* women with NSCLC (p = 0.958; FDR p = 0.966), or healthy men *vs.* men with NSCLC (Figure 2(A); p = 0.573; FDR p = 0.690) in univariate analyses, they are slightly higher in healthy men than healthy women (p = 0.022; FDR p = 0.051). In addition, sHER2 concentrations trend towards being higher in women with NSCLC compared to healthy women (Figure 2(B); p = 0.052; FDR p = 0.098).

**Table 2:** Serum sHER2 concentrations by tumor histology. Serum sHER2 concentrations are compared between lung cancer cases with squamous cell carcinomas *vs.* adenocarcinomas by gender, and healthy men and women (*i.e*., controls) *vs.* patients with squamous cell carcinomas and adenocarcinomas by gender.

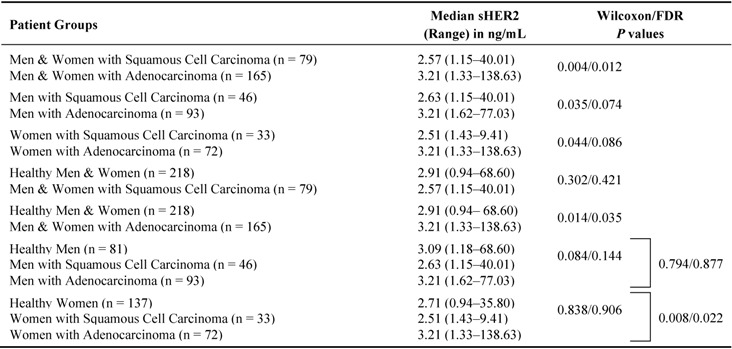

**Table 3:** Unadjusted relative risk of tumor histological subtype associated with serum sHER2 concentration. Unadjusted odds ratios (OR) and 95% confidence intervals (95% CI) comparing adenocarcinomas *vs.* squamous cell carcinomas are shown for quintiles of serum sHER2 concentration by gender.

Quintiles of Serum sHER2	OR (95% CI) for Women	OR (95% CI) for Men
≤1.85 ng/mL	referent	1.01 (0.57, 1.78)
2.40 ng/mL	1.48 (1.09, 2.02)	1.49 (0.79, 2.82)
3.00 ng/mL	2.06 (1.16, 3.66)	2.08 (0.94, 4.62)
3.65 ng/mL	2.77 (1.23, 6.22)	2.79 (1.05, 7.74)
≥6.60 ng/mL	6.74 (1.48, 30.67)	6.78 (1.36, 33.79)

**Figure 3:**
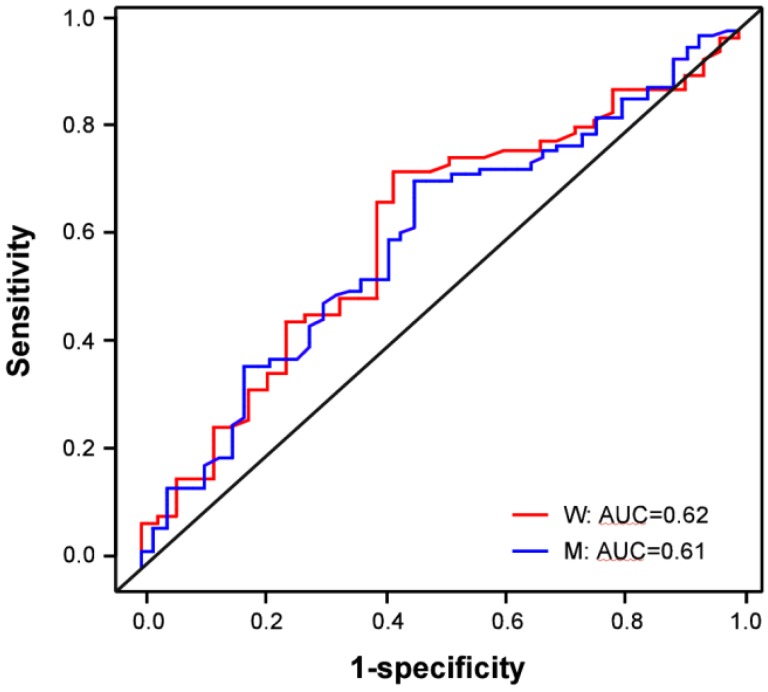
Receiver operating characteristic curves for log serum sHER2 concentrations of adenocarcinoma *vs.* squamous cell carcinoma.ROC curves for log-transformed sHER2 concentrations are shown comparing patients with adenocarcinoma *vs.* SCC for men (blue ROC curve labeled M) and women (red ROC curve labeled W), respectively.

Further comparison of NSCLC cases by histological subtype shows that serum sHER2 concentrations are slightly higher in patients with adenocarcinoma than SCC among men (Figure 2(C); Table 2; p = 0.035; borderline significance, FDR p = 0.074), among women (Figure 2(D); p = 0.044; borderline significance, FDR p = 0.086), and both genders combined (p = 0.004; FDR p = 0.012). Although sHER2 concentrations do not differ between healthy controls and patients with SCC among men (borderline significance, p = 0.084; FDR p = 0.144) or among women (p = 0.838; FDR p = 0.906), sHER2 concentrations are higher in women (p = 0.008; FDR p = 0.022) but not in men with adenocarcinoma (p = 0.794; FDR p = 0.877) compared to healthy controls of the same gender. Logistic regression models and ROC curves (Figure 3) demonstrate that unadjusted for age, log-transformed sHER2 concentrations have a statistically significant, but weak ability to differentiate patients with adenocarcinoma from SCC among men (AUC = 0.610) and women (AUC = 0.623). Furthermore, both men and women with high serum sHER2 concentrations have higher risk of having adenocarcinoma than SCC (Table 3).

### 3.3. sHER2 is a Weak, Independent Discriminatory Biomarker of Adenocarcinoma

**Figure 4:**
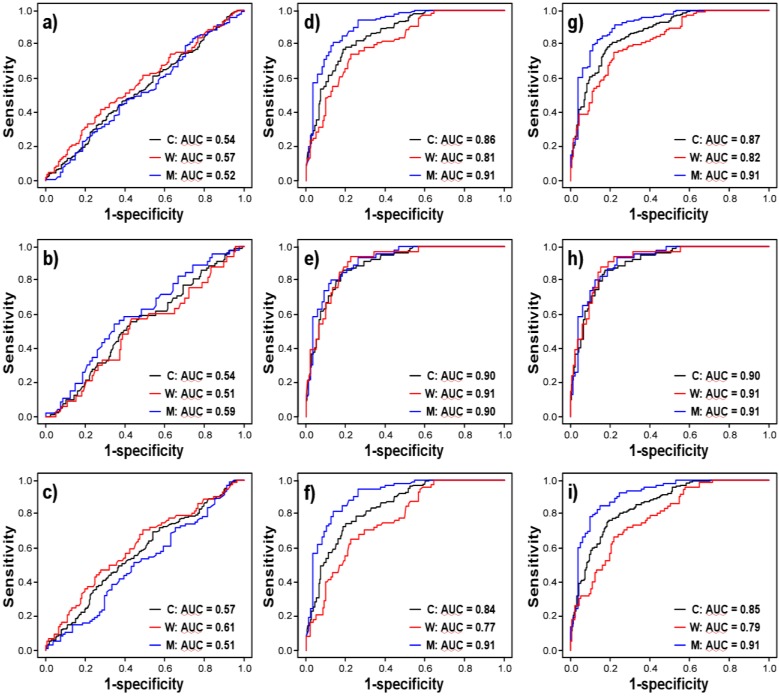
Receiver operating characteristic curves of lung cancer cases *vs.* controls. ROC curves are shown comparing all cancer cases *vs.* healthy controls (**a**, **d**, **g**), SCC *vs.* healthy controls (**b**, **e**, **h**), and adenocarcinoma *vs.* healthy controls (**c**, **f**, **i**). ROC curves are shown for a reduced model of log-transformed sHER2 concentrations alone (a, b, c), a model of age alone (d, e, f), and a model including both age and log-transformed sHER2 concentrations (g, h, i) for both men and women combined (black ROC curves labeled C), men only (blue ROC curves labeled M), and women only (red ROC curves labeled W).

Univariate logistic regression models show that both age (p < 0.0001; FDR p < 0.0005) and gender (p < 0.0001; FDR p < 0.0005), but not log-transformed sHER2 concentrations (p = 0.191; FDR p = 0.298) are associated with NSCLC when adenocarcinoma and SCC are combined (Table 4). Likewise, age (p < 0.0001; FDR p < 0.0005) and gender (p = 0.002; FDR p = 0.007), but not sHER2 concentrations (p = 0.290; FDR p = 0.415) are associated with SCC. In contrast, sHER2 concentrations (p = 0.026; borderline significance, FDR p = 0.057), age (p < 0.0001; FDR p < 0.0005), and gender (p = 0.0003; FDR p = 0.0012) are associated with adenocarcinoma. Multivariate logistic regression models further demonstrate that log-transformed sHER2 remains independently associated with adenocarcinoma (p = 0.022; FDR p = 0.051), but not SCC (p = 0.891; FDR p = 0.945) or all NSCLC tumors combined (borderline significance, p = 0.082; FDR p = 0.144) when adjusted for age and gender. ROC curves derived from univariate logistic regression models illustrate that age is a strong discriminator of healthy controls from all NSCLC tumors combined (AUC = 0.858), as well as cases diagnosed with SCC (AUC = 0.895) or adenocarcinoma (AUC = 0.840), potentially reflecting ascertainment bias; while gender is a weak discriminator of all NSCLC tumors combined (AUC = 0.594), SCC (AUC = 0.601) or adenocarcinoma (AUC = 0.591) (Table 4 and Figure 4). In contrast, log-transformed sHER2 alone does not discriminate healthy controls from all NSCLC cases combined (AUC = 0.537) or SCC (AUC = 0.539), but weakly discriminates adenocarcinoma (AUC = 0.573). When mutually adjusted for each other, sHER2, age, and gender yield 85.9% accuracy (AUC) to discern healthy controls from patients with lung adenocarcinoma across all cut-off thresholds of sHER2 (Table 4). Notably, the ability to discern healthy controls from patients with adenocarcinoma is contributed mainly by age rather than sHER2 or gender (AUC = 0.840 with age alone *vs*. AUC = 0.859 with age, gender, and sHER2 combined). These ROC curves further demonstrate that gender is an important confounder of age for discerning healthy controls from patients with adenocarcinoma, but not SCC (compare Figure 4(F) to 4(E) and 4(I) to 4(H)), whereby being male is a stronger determinant of adenocarcinoma than being female. For example, ROC curves that include age and sHER2 (or age alone, Figure 4(F)) show statistically significantly better discrimination of healthy controls *vs.* patients with adenocarcinoma among men (AUC = 0.91) than among women (AUC = 0.78) (Figure 4(I)); notably, the sensitivity to detect adenocarcinoma is 60% for men, but only 30% for women at 95% specificity (Figure 4(I)). Adjusted for age and gender, the risk of adenocarcinoma increase with higher serum sHER2 concentrations (Table 5). For example, adjusted for age, the odds of adenocarcinoma are 3.95 (95% CI: 1.22, 12.81) among women and 7.93 (95% CI: 2.26, 27.82) among men at a value of >6.60 ng/mL sHER2 compared to the referent value of <1.85 ng/mL. The confounding effect of gender on age is not observed in logistic regression models that include age and sHER2 (or age alone) for patients with SCC (Figure 4(E)); *i.e.*, men (AUC = 0.91) and women (AUC = 0.91) with SCC are discriminated equally well from healthy controls of the same gender by age alone (Figure 4(H)). Finally, no evidence of effect modification by age or gender on the association between serum sHER2 and NSCLS, adenocarcinoma, or SCC is observed in this dataset (not shown). Taken together, these analyses show that age and gender are classifiers of NSCLC, SCC, and adenocarcinoma; as well as confounders of the association between sHER2 and adenocarcinoma in this dataset. Nevertheless, serum sHER2 remains a weak, statistically significant, independent discriminatory biomarker of lung adenocarcinoma, but not of SCC, after adjusting for age and gender.

**Table 4:** Logistic regression models of non-small cell lung cancer (NSCLC) with age, gender, and log-transformed serum sHER2 concentrations as covariates. Univariate and multivariate logistic regression models are shown of men and women with lung cancer compared to healthy men and women (*i.e*., controls) for all lung cancer cases combined, and stratified by tumor histological subtype.

**Univariate Logistic Regression Models**						
**Patient Groups** **Parameters**		**Maximum Likelihood Estimate**	**Standard Error**	**Wald *Χ^2^***	***Χ^2^*** **/FDR** ***P*** **value**	**AUC**
All Lung Cancer Cases *vs.* Controls						
	Age	0.106	0.010	125.216	<0.0001/<0.0005	0.858
	Gender	−0.766	0.187	16.728	<0.0001/<0.0005	0.594
	Log sHER2	0.429	0.328	1.711	0.191/0.298	0.537
Squamous Cell Carcinomas *vs.* Controls						
	Age	0.129	0.016	66.308	<0.0001/<0.0005	0.895
	Gender	−0.818	0.265	9.499	0.002/0.007	0.601
	Log sHER2	−0.565	0.534	1.119	0.290/0.415	0.539
Adenocarcinomas *vs.* Controls						
	Age	0.098	0.010	95.433	<0.0001/<0.0005	0.840
	Gender	−0.741	0.207	12.788	0.0003/0.0012	0.591
	Log sHER2	0.777	0.350	4.937	0.026/0.057	0.573
**Multivariate Logistic Regression Models**						
**Patient Groups** **Parameters**		**Maximum Likelihood Estimate**	**Standard Error**	**Wald *Χ^2^***	***Χ^2^*** **/FDR** ***P*** **value**	**AUC**
All Lung Cancer Cases *vs.* Controls						
	Age	0.112	0.010	121.132	<0.0001/<0.0005	
	Gender	−0.831	0.252	10.831	0.001/0.004	
	Log sHER2	0.801	0.461	3.024	0.082/0.144	0.877
Squamous Cell Carcinomas *vs.* Controls						
	Age	0.140	0.018	63.664	<0.0001/<0.0005	
	Gender	−1.416	0.386	13.462	0.0002/0.0009	
	Log sHER2	0.112	0.817	0.019	0.891/0.945	0.917
Adenocarcinomas *vs.* Controls						
	Age	0.102	0.011	93.161	<0.0001/<0.0005	
	Gender	−0.697	0.267	6.807	0.009/0.024	
	Log sHER2	1.080	0.472	5.235	0.022/0.051	0.859

**Table 5:** Age-adjusted relative risk of adenocarcinoma associated with serum sHER2 concentration. Age-adjusted odds ratios (OR) and 95% confidence intervals (95% CI) comparing healthy men and women (*i.e*., controls) *vs.* patients with adenocarcinoma are shown for quintiles of serum sHER2 concentration by gender.

Quintiles of Serum sHER2	OR (95% CI) for Women	OR (95% CI) for Men
≤1.85 ng/mL	referent	2.01 (1.19, 3.39)
2.40 ng/mL	1.32 (1.04, 1.68)	2.66 (1.52, 4.66)
3.00 ng/mL	1.69 (1.08, 2.64)	3.38 (1.74, 6.57)
3.65 ng/mL	2.08 (1.11, 3.90)	4.18 (1.90, 9.21)
≥6.60 ng/mL	3.95 (1.22, 12.81)	7.93 (2.26, 27.82)

## 4. Discussion

Steroid hormones have been implicated in the regulation of HER2 expression; however, the age and gender specific effects of steroids on HER2 expression, as well as on sHER2 shedding is not completely understood. In human breast cancer cells *in vitro*, estradiol is a potent inhibitor of HER2 expression and sHER2 shedding [41,42,43,44]; whereas, progesterone positively regulates HER2 expression [45]. In contrast, neither estrogen nor progesterone regulates HER2 expression in ovarian cancer cells [46]. Among men with prostate cancer, high HER2 expression has been associated with androgen ablation therapy and androgen independent prostate carcinoma [47]. Among healthy women, oral contraceptive use and hormone replacement therapy have been associated with lower serum sHER2 concentrations [48]. In pregnant women, serum sHER2 concentrations are lower during the first and second trimester in pregnant women, which is when estrogen concentrations increase, but are higher during the third trimester [49,50,51]. Finally, elevated serum sHER2 concentrations among women with preeclampsia *vs.* a normal pregnancy suggest the possibility of fetomaternal transfer during the third trimester of pregnancy [49,52]. Together, these observations suggest that steroid hormones may regulate both the biosynthesis and shedding of sHER2 into circulatory fluids. Our observations that sHER2 concentrations decrease with age in healthy men, and that age and gender confound the relationship between sHER2 concentrations and lung adenocarcinoma are consistent with hormonal regulation of HER2 expression and/or shedding. These observations underscore the importance of adjusting for age, gender, and other potential confounding variables that may affect steroid hormone biosynthesis, such as body mass and smoking [53,54], when evaluating the utility of sHER2 for the risk assessment, early detection, and diagnosis of adenocarcinoma using epidemiological study designs and multivariate statistical methods.

Limitations and caveats of this retrospective study include: (a) an inability to assess the effect of long term storage at −80 °C on serum sHER2 stability, (b) an inability to assess potential differences in serum sHER2 concentrations between non-small cell adenocarcinomas compared to large or small cell carcinomas; (c) an inability to assess potential differences in serum sHER2 concentrations by disease stage or tumor grade; (d) lack of data on potential confounders or effect modifiers of sHER2 concentrations such as smoking status, body mass, personal and family cancer history, carcinogen exposure, menstrual cycle phase, parity, and exogenous hormone use among women; (e) potential ascertainment and cohort bias of the control group, which was not selected using the same enrollment criteria (e.g., age and smoking status) or at random during the same time period from the source population as the cases; and (f) an inability to assess the risk of developing lung adenocarcinoma among asymptomatic individuals with pre-diagnostic serum samples. Given these caveats and limitations, it will be important to conduct well-designed transitional studies to assess the effects of storage at −80 °C on serum sHER2 concentrations, and prospective case-control studies to further validate sHER2 as a weak, independent biomarker of lung adenocarcinoma using multivariate models. Such studies should include incident cases of lung adenocarcinoma, other malignant and benign lung neoplasms, and healthy controls selected randomly from the study population.

Several reports indicate that serum sHER2 concentrations have 5–64% sensitivity to detect lung cancer of various histological subtypes using cut-off thresholds that correctly classify healthy controls with 95% specificity [23,32,33,34,35,36]. Sensitivity was reported to be higher for patients with late stage lung cancer and for patients with adenocarcinoma compared to SCC, large cell carcinoma, and small cell carcinoma [23,32,34,35]. In general, these studies were small, used disparate immunoassays, or used previously established cut-off thresholds from an external control study group [23]. Additionally, most of these studies did not control for confounding by age, gender, and other variables such as smoking by using a matched, case-control study design or by adjusting for confounding in an unmatched, case-control study design with multivariate logistic regression methods [23,32,34,35,36]. Notably, the highest reported sensitivity (64%) and specificity (95%) of sHER2 concentrations to discern healthy controls from lung cancer cases was reported for a small age-, gender-, ethnic group-, and smoking-matched case-control study [33]. That age might be a potential confounder or effect modifier of the association between sHER2 and cancer is supported by our observation that sHER2 concentrations decrease with age in healthy men, and by a previous report showing that sHER2 concentrations increase with age in healthy women independently of menopausal status [55]. Here, it is observed that sHER2 concentrations are higher in primary NSCLC patients with adenocarcinoma than SCC, thus confirming that biological differences exist between these histological subtypes. Moreover, we report that the ability of sHER2 concentrations to discern healthy controls from patients with adenocarcinoma, but not SCC, is indeed confounded by age and gender in our dataset (Table 4 and Figure 4). When mutually adjusted for each other, sHER2, age, and gender yield 85.9% accuracy to classify controls from primary adenocarcinoma patients (Table 4). However, the strength to discern healthy controls from patients with adenocarcinoma is contributed mainly by age, not sHER2 and gender (AUC = 0.840 with age alone *vs*. AUC = 0.859 with age, gender, and sHER2 combined). The accuracy to discern adenocarcinoma patients from controls with age-adjusted sHER2 concentrations is 91% among men, but only 78% among women (Figure 4(I)), suggesting that additional confounders may be influencing the association between sHER2 and adenocarcinoma among women. Notably, at 95% specificity, the sensitivity to detect adenocarcinoma is 60% for men and 30% for women; these statistics are similar to those reported by Brandt-Rauf and colleagues, who used a matched case-control study design to control for confounding by age, gender, ethnicity, and smoking [33]. Importantly, the data reported here show that sHER2 is a weak, but statistically significant, independent classifier of adenocarcinoma. Although sHER2 is not a strong, stand-alone classifier of adenocarcinoma, its independence of age and gender may make sHER2 a useful covariate (with age, gender, and other biomarkers) in multivariate screening and diagnostic models of lung adenocarcinoma.

Risk prediction models that include smoking are useful for advising patients about their propensity to develop lung cancer. However, while smoking is a well-known risk factor for lung cancer, considered alone, smoking history has limited ability to predict which smokers will develop lung cancer. Other contributing epidemiological, clinical, and biological (*i.e*., biomarkers) factors may improve lung cancer risk prediction among smokers and even non-smokers [56,57]. Several groups have developed lung cancer risk models using the variables of age, smoking history, carcinogen exposure, and family history [58,59,60,61,62,63,64]. However, few of these studies included serum biomarkers, despite our understanding that biomarkers represent promising risk assessment tools [56,57,59,64]. Recently, Spitz and colleagues modestly improved their risk model, which included environmental tobacco smoke, family cancer history, dust exposure, prior respiratory disease, hay fever, and smoking history variables by adding two *in vitro* markers of DNA repair capacity (host-cell reactivation and mutagen sensitivity) [65]. Adding these markers significantly improved the model’s accuracy to discriminate between lung cancer cases and controls from 67% to 70% among former smokers (p = 0.006), and from 68% to 73% among current smokers (p = 0.0048). Similarly, Prindiville and colleagues found that sputum samples characterized by moderate to worse cytological atypia defined a cohort of high-risk patients who were at increased risk of developing lung cancer [66]. Notably, we observe that, adjusted for age, the relative risk (odds) of adenocarcinoma is 3.95 (95% CI: 1.22, 12.81) among women and 7.93 (95% CI: 2.26, 27.82) among men with high sHER2 concentrations (≥6.60 ng/mL) compared to women or men with low sHER2 concentrations (≤1.85 ng/mL; Table 5). These data are consistent, but not conclusive, with a role for serum sHER2 as a biological marker of risk for primary non-small cell lung adenocarcinoma.

Cancer treatment is undergoing a paradigm shift from the use of broad-spectrum cytotoxic chemotherapeutics to biologically targeted therapeutics [67], including HER-targeted drugs [68]. The HER family of receptors, EGFR/HER1, HER2, HER3, and HER4 are rational targets for anti-cancer drugs because they activate complex signal transduction pathways that lead to tumor cell proliferation, survival, and metastasis [69]. In patients with NSCLC, EGFR-targeted RTK inhibitors, such as gefitinib and erlotinib, have produced objective response rates in about 10% of patients with advanced stage disease and modest improvements in patient survival [70,71,72]. Clinical trials with HER2-targeted monoclonal antibodies, trastuzumab and pertuzumab, also have shown efficacy in some NSCLC patients [70,73,74]; however, trials with trastuzumab have been insufficiently powered to determine whether NSCLC patients with *HER2* gene amplification or overexpression as determined by immunohistochemistry may benefit from treatment [75,76]. Notably, responsiveness and efficacy toward HER-targeted therapeutics is dependent on the selection of patients who present with the active target of choice, and accurate methods to identify these patients [77,78]. Here, we observe that serum sHER2 concentrations detected by ELISA are higher in a subset of primary NSCLC patients with adenocarcinoma (adjusted for age, 60% of men and 30% of women had sHER2 concentrations above the 95th cut-off threshold for healthy controls), suggesting that sHER2 may warrant investigation as a theragnostic biomarker to select a subset of patients with lung adenocarcinoma for treatment with HER2-targeted drugs.

## 5. Conclusions

In summary, we have observed that serum sHER2 concentrations are higher in a subset of patients with primary adenocarcinoma compared to patients with SCC or healthy controls, and weakly, but independently associated with adenocarcinoma even after adjusting for confounding by age and gender. These results are consistent with the hypothesis that sHER2 may have some limited clinical utility as a serum biomarker of non-small cell lung adenocarcinoma, if properly adjusted for confounding by age, gender, and variables that affect circulating and tissue steroid hormone concentrations (e.g., body mass, smoking, oral contraceptive use, and hormone replacement therapy) or circulating steroid hormones themselves. We conclude that even though serum sHER2 is not a strong, stand-alone discriminatory biomarker of adenocarcinoma, sHER2 may be a useful, independent covariate in multivariate regression models that warrants further investigation for the risk assessment, screening, and diagnosis of lung adenocarcinoma in well-designed epidemiological studies that control for confounding by age, gender, and other demographical, epidemiological, behavioral, and environmental variables using multivariate regression methods or a matched case-control study design.
